# Supplementary material for: Optimizing reliability of RBC signal oscillation measures from hyperpolarized 129Xe MRI
Source: PLoS One. 2025 Aug 26;20(8):e0331113. doi: 10.1371/journal.pone.0331113 (PMC12380273; doi:10.1371/journal.pone.0331113)
Supplement: Attachment — Submitted filename: Response_to_Reviewers.docx [file pone.0331113.s001.docx]

We thank the reviewers for their feedback, which has enabled us to strengthen our manuscript. All changes in response to reviewer comments are tracked and commented using the shorthand R for reviewer and C for comment.

Editor Comments:

**Comment 1:** Please ensure that your manuscript meets PLOS ONE's style requirements, including those for file naming. The PLOS ONE style templates can be found at

[https://journals.plos.org/plosone/s/file?id=wjVg/PLOSOne_formatting_sample_main_body.pdf](https://url.us.m.mimecastprotect.com/s/nExdCgJGQNhly4ym4u3s8i4K4dX?domain=journals.plos.org) and

[https://journals.plos.org/plosone/s/file?id=ba62/PLOSOne_formatting_sample_title_authors_affiliations.pdf](https://url.us.m.mimecastprotect.com/s/vvpaCjRkONhjmZm3ZU1t9imQUfT?domain=track.editorialmanager.com)

**Response:** We have formatted our manuscript according to the shared style templates.

**Comment 2.** Please expand the acronym “NIH” (as indicated in your financial disclosure) so that it states the name of your funders in full.
This information should be included in your cover letter; we will change the online submission form on your behalf.

**Response:** We have expanded “NIH” into National Institutes of Health and also include this funding information in our resubmission cover letter.

**Comment 3.** Thank you for stating the following in the Competing Interests/Financial Disclosure section:
I have read the journal's policy and the authors of this manuscript have the following competing interests: Peter Niedbalski is a consultant for Polarean Imaging, Plc.

We note that one or more of the authors are employed by a commercial company: Polarean Imaging, Plc

a. Please provide an amended Funding Statement declaring this commercial affiliation, as well as a statement regarding the Role of Funders in your study. If the funding organization did not play a role in the study design, data collection and analysis, decision to publish, or preparation of the manuscript and only provided financial support in the form of authors' salaries and/or research materials, please review your statements relating to the author contributions, and ensure you have specifically and accurately indicated the role(s) that these authors had in your study. You can update author roles in the Author Contributions section of the online submission form.

Please also include the following statement within your amended Funding Statement.
“The funder provided support in the form of salaries for authors [insert relevant initials], but did not have any additional role in the study design, data collection and analysis, decision to publish, or preparation of the manuscript. The specific roles of these authors are articulated in the ‘author contributions’ section.”
If your commercial affiliation did play a role in your study, please state and explain this role within your updated Funding Statement.

b. Please also provide an updated Competing Interests Statement declaring this commercial affiliation along with any other relevant declarations relating to employment, consultancy, patents, products in development, or marketed products, etc.

Within your Competing Interests Statement, please confirm that this commercial affiliation does not alter your adherence to all PLOS ONE policies on sharing data and materials by including the following statement: "This does not alter our adherence to PLOS ONE policies on sharing data and materials.” (as detailed online in our guide for authors [http://journals.plos.org/plosone/s/competing-interests](http://url.us.m.mimecastprotect.com/s/36FKCkRlONhnBoBrot8uxiGr-qs?domain=journals.plos.org)) . If this adherence statement is not accurate and there are restrictions on sharing of data and/or materials, please state these. Please note that we cannot proceed with consideration of your article until this information has been declared.

Please include both an updated Funding Statement and Competing Interests Statement in your cover letter. We will change the online submission form on your behalf.

**Response:** We have provided revised statements in our cover letter as requested. We note that Dr. Niedbalski is not an employee of Polarean Imaging, Plc but only receives personal consulting fees unrelated to the present manuscript. We believe that our revised statements adhere to the PLOS ONE guidelines and better describe Dr. Niedbalski’s role, but we are happy to work with you to revise as appropriate.

**Comment 4.** Please include your full ethics statement in the ‘Methods’ section of your manuscript file. In your statement, please include the full name of the IRB or ethics committee who approved or waived your study, as well as whether or not you obtained informed written or verbal consent. If consent was waived for your study, please include this information in your statement as well.

**Response:** We have added our complete ethics statement in the Methods section and included a statement that written informed consent was acquired from all participants.

**Comment 5**. If the reviewer comments include a recommendation to cite specific previously published works, please review and evaluate these publications to determine whether they are relevant and should be cited. There is no requirement to cite these works unless the editor has indicated otherwise.

Response: Reviewers did not request that we cite other works.


Reviewer Comments

**REVIEWER 1**

**Summary:** The authors describe an assessment of repeatability/reliability for RBC oscillation imaging acquired as part of the gas exchange Xe-MRI acquisition. They acquired data in specific populations which are expected to represent the majority of the signal variations observed in patients. They also compare multiple RBC oscillation analysis methods to provide insight into which may be better suited for applications to disease with the goal to optimize the methods. Overall, the manuscript is well done but could use some minor improvements to fully support their findings.

**Response:** We thank the reviewer for their positive assessment of our manuscript.
Comments:
1. Line 118: I suppose this TR is the TR between the same compartments and not between RF pulses. Please clarify.

**Response 1**: This interpretation is correct and has been clarified in the revised document

2. Line 139: The 20% threshold method does not appear to be assessed/considered here. Has this method already been shown inferior? If so, how/where? If not, consider including it for reference.

**Response 2:** To our knowledge, this method has not explicitly been shown to be inferior. However, as the developer of this 20% method (Senior author P. Niedbalski), there are clear downsides to that method. It requires a peak-by-peak scaling of data to correct for the different amplitudes of oscillations observed throughout the breath hold. It also is very susceptible to incorrect binning due to noise spikes. While this is less of a concern for healthy volunteers, PAH patients with suppressed oscillations are likely to have noise from intermediate portions of the cardiac cycle that end up being binned to high or low keys. With these clear limitations, we do not feel that it is necessary to explicitly demonstrate inferiority, particularly given a lack of gold standard to compare these measures to. We have added a sentence to the methods to provide some justification of this.

3. Line 147: To detrend the dissolved signal, have the authors considered normalizing by the gas? I believe this would be more robust as they will be significantly correlated. It won’t contain the downstream magnetization decay, but would those projections not be discarded anyway?

**Response 3**. This is a good idea and something that we had considered. In our experience, this provides similar performance for detrending. However, it has a minor downside in that variations in the gas phase signal, for example due to early exhalation, are mirrored in the dissolved signal. Scaling dissolved signal by the gas in this case may smooth out that variation and fail to signal that there is a significant change in lung inflation partway through the scan (we note that all participants in this study had good breath-hold compliance). In the absence of clear benefit to scaling by the gas signal, we opted to maintain continuity and use a double exponential fit.

4. Line 150: What is mean by a multi-component sine fit? Sum of two sine waves?

**Response 4:** We actually used a sum of 5 sine waves to do this fit, the purpose being to have terms that could accommodate variations in signal intensity throughout the breath-hold. We have clarified this in the methods section.

5. Line 151: How was one cardiac cycle determined? From the measured heart rate or the data?

**Response 5:** We have clarified in the text that this was determined from the data.

6. Line 157: Have the authors considered calculating by median rather than mean? It would be more robust to outliers, but I am not sure if that’s an issue.

**Response 6:** This is an interesting suggestion that we had not considered. Generally speaking, in this dataset we did not have issues with serious outliers that necessitate the use of a median. To confirm this, we re-ran our analysis using median rather than mean and found oscillation amplitude to be identical whether scaling by mean or median.

7. Line 201: I was initially confused by the underscore v in the equations before realizing it was for voxel. Would it make more sense to not have it since they all are 3D maps? Or use (x,y,z) instead?

**Response 7:** Thank you for pointing out this source of confusion. We like the suggestion of (x,y,z) and we have edited the text. We note that we believe it is helpful to emphasize that these maps are on the voxel level.

8. Line 218: What is the reason behind using only distance and not also the even more common Dice coefficient? I assume its due to Dices poor performance with small/thin structures, but it would be useful to know.

**Response 8:** This is exactly right and we have clarified this in the text.

9. Line 233: With the reduced 6-week reliability performance, was it confirmed that none of the patients had a change in treatment/medication or other reasons for varying hypertension.

**Response 9.** This is a good suggestion, and we have gone back and reviewed all participants. In this review, we identified that one participant was hospitalized between baseline and 6-weeks, and one participant was diagnosed with COVID-19. All other participants were stable and had no changes in medication. We re-ran our analysis, omitting these 2 participants. This changed the numerical value of correlation coefficients, but the take-home messages remained unchanged. We have updated the manuscript with this new analysis.

10. Line 241: Can you provide insight into why reliability was higher for PAH? That goes against my intuition of lower signal and lower oscillations being more prone to error.

**Response 10.** We agree that this is unexpected, and we do not fully understand why this is. The one thing we can say is that, counterintuitively, the reliability of oscillation measurements don’t seem to be strongly related to oscillation quality. We have added some description of this in the discussion, but we agree that this is unexpected and likely warrants further study.

11. Line 255: The success of the bandpass over the lowpass suggests a low frequency component being present. Would comment 3 help with this?

**Response 11.** In some participants, there is some slight low frequency component that is visible to the naked eye, but the more significant impact was high frequency noise data. We note that we did not implement a low pass filter in the present work, though it may be that scaling by gas and using a low pass filter could provide similar performance to a bandpass filter. However, a bandpass filter performs quite well in these data, so this additional analysis does not seem to be warranted.

12. Line 277: How was SNR of a single frequency measured?

**Response 12:** SNR was measured as the maximum of the peak divided by the mean noise (measured at the edge of the frequency range). We have added clarification to the methods section.

13. Line 300: In the model, HR difference was the true difference and not the abs() like mentioned earlier, correct?

**Response 13:** The absolute value was used for all HR difference calculations.

14. Line 301: Could you clarify how HR difference was significant despite a p=0.49?

**Response 14:** This was a typo and should have read p = 0.049. This has been corrected.

15. Line 331: Weren’t these supposed to be anova’s? If so, were they significant before assessing the post hocs?

**Response 15:** Thank you for pointing out this source of confusion. We have clarified in the methods that post-hoc testing was only performed if ANOVA returned a significant p value.

16. Line 333:P=0.09 for both PAH vs healthy and PAH vs SSc?

**Response 16:** We have clarified that p = 0.09 for PAH vs. healthy. PAH vs. SSc was reported in the previous sentence as p<0.001, though we acknowledge that the wording was unclear in the original manuscript.

17. Line 382: Why was mse and distance not assessed for the 6-week tests like the same day?

**Response 17:** We have added details about MSE and Average Distance for 6-week tests.

18. Line 399: I may have missed it, but I don’t see anywhere where the effect of data quality on each method was performed.

**Response 18:** We had previously examined whether there was an association between SSIM and oscillation quality, finding no correlation. However, this was not reported in the original manuscript. We have expanded this analysis to SSIM, MSE, and average distance for each of the oscillation bins. Generally, there were few significant correlations, but what was correlated is now reported in an additional paragraph near the end of the results section. We have also slightly expanded our discussion of this based on these new results.

19. Line 432: I would like to see the values and stats for the gradients since they seem important.

**Response 19.** We have added a figure (Fig 8) that shows anterior to posterior and apical to basal gradients for healthy volunteers. Only healthy volunteers are shown because disease may change the natural regional variation in signal. We have added slightly to our presentation of the results to fully discuss this figure.

20. Line 437: Did voxel wise reduce the variation of the signal within the lung or did it just make them appear more physiologically accurate?

**Response 20.** This is an excellent question and one that we should have examined from the beginning. As a coarse measure of variation, we calculated the mean coefficient of variation of oscillation amplitude in the lungs of healthy volunteers (as pathologically driven factors will likely increase variation in patients with lung disease). Coefficient of variation was lowest in the mean-scaled approach, and highest for multi-key approach. We have added this additional analysis to the results and discussion sections.

21. Line 473: The multi-key approach with phase-based scaling seems to do just as well so is this true?

**Response 21.** A fair point. We have added a sentence noting that combining amplitude and phase information appears to recover disease sensitivity.

22. Fig 7: This may be in the referenced paper, but is there any insight into why the phase maps are not continuous? Is that an artifact of the method or is it expected to be physiological? I could see it having sharp transitions for regional “up-stream” issues but it all seems to be very digitized in the phase dimension.
**Response 22.** Unfortunately, this feature was not discussed in the original Pilgrim-Morris paper, despite that group also presenting very digitized phase maps. We believe that these sharp transitions are likely a combination of an artifact of the method and real physiology. 1) Artifact of the method: Based on the way phase maps are calculated, phase can only be calculated at discrete steps of ~3 deg (0.05 rad), which can lend to some appearance of digitization.

2) Real physiology: Because different regions of the capillary bed are fed by larger upstream vessels, occlusions or differing flow characteristics in an upstream vessel could lead to uniformly altered phase in a large part of the lungs. Thus, sharp edges in the phase map are not totally unexpected, particularly in patients with pulmonary vascular disease.

More work is needed in this space. A possible next step would be to examine the regions of differing phase in the context of a vascular segmentation from CT. By examining whether a region of uniform phase appears to be fed by a single upstream vessel, this could begin to provide stronger evidence for the physiological validity of these phase maps. Such analysis would be quite involved and is beyond the scope of the present work.

23. Fig 9: I assume these metrics are all for the raw multi-key oscillations? I think it would be useful to clarify and/or include the scaled version. Also, if it’s the raw values, you should likely use a normalized version to not penalize the multi-key method which produces higher oscillation percentages.

**Response 23.** This is a glaring oversight on our part. While SSIM and bin comparisons using average distance are going to be unaffected by the voxel intensities, MSE is impacted by this. As such, our original MSE calculation severely penalized the multi-key method and led to an unfair comparison. We have redone this calculation, first converting each oscillation amplitude map to a modified z-score prior to image registration and MSE calculation. Details of this additional step have been added to the methods section, Fig 9 has been updated, and values have been updated throughout the manuscript.

Reviewer #2:

**Summary:**
Periodic oscillations in the signal from 129Xe dissolved in the pulmonary capillary red blood cells (RBCs) originating from changes in capillary blood volume over the cardiac cycle have recently emerged as a potential biomarker of cardiopulmonary disease. The amplitude of the oscillations can be mapped regionally from 3D radial dissolved-phase 129Xe imaging data using a post-acquisition keyhole reconstruction. This manuscript aimed to optimise the oscillation mapping method. The authors present a comparison of three published variations on the keyhole mapping method and evaluate their same-day and 6-week repeatability. Each method was able to detect differences in the oscillation amplitude of patients with pulmonary arterial hypertension and systemic sclerosis. This is an important contribution to the limited literature on RBC oscillation mapping and has several strengths, including:
• Implementation of the oscillation mapping technique to two patient groups with reduced and enhanced oscillation amplitude respectively, including first evaluation of oscillation mapping in SSc patients
• Thorough evaluation of different binning algorithms and normalisation techniques
• Establishment of an interesting relationship between the oscillation amplitude from the two-key method and the amplitude and phase from the multi-key method

I have some general comments followed by some minor, more specific, comments.

**Response:** We thank the reviewer for their recognition of the strengths of our manuscript.


Major Comments:
1. The authors mention that an advantage of the multi-key method is its ability to map both oscillation amplitude and phase, however only oscillation amplitude is analysed here. The RBC oscillation phase is new and relatively unexplored, so it would be interesting to also include these results, i.e. repeatability of phase, comparison between the groups.

**Response 1.** We have added some description of the repeatability of phase and how phase measures compare between groups.

2. The paper does a good job of comparing the three mapping methods in terms of their reliability, but some further detail about the differences between the 2-key and multi-key methods is needed. For example, what are the interpretation and implications of the negative amplitude values obtained from the 2-key approach?

**Response 2.** We have added a paragraph to the discussion section describing some of the features beyond reliability that may contribute to the choice of a 2-key vs. a multi-key approach.

3. It is interesting that the reliability of the oscillation maps was not strongly affected by oscillation quality. I can see that for the 2-key method, as long as the maxima and minima can still be identified, noisy oscillations might not cause too much of a problem. However, for the multi-key method, wouldn’t the points chosen by the sliding window become out of sync with each other in cases such as in Figure 4A-C, so that different points in the cardiac cycle are being sampled? How does this impact the phase maps? Do the authors think there is a cut-off for SNR or any other quality metric, or is it okay to perform oscillation mapping regardless of oscillation quality?

**Response 3.** We completely agree that this was a surprising finding. Ultimately, it appears that the bandpass filtering method of binning oscillations is effective enough at identifying oscillation extrema that oscillation quality is not a large concern. The multi-key method is based on the maxima detected for the 2-key method. Thus, if the 2-key method is identifying peaks accurately, the multi-key method should be sampling the cardiac cycle as expected. Prior to completing the analysis for this manuscript, we would have definitely said there was some quality cutoff after which oscillation imaging should not be performed. One of our goals in this project was to identify such a cut-off, ideally using quantifiable methods such as SNR, full width at half maximum, or similar. However, the data we present in this manuscript just don’t suggest that there is such a cut-off. It is very counterintuitive, and we would be very open to new data providing evidence that quality makes a difference for reliability. We can only base our conclusions on our data, though, so to this point, we have to conclude that oscillation quality does not seem to strongly determine the reliability of these oscillation measures.

Minor Comments:
1. Abstract: add a sentence to summarise how the oscillation mapping method was optimised using healthy volunteer data

**Response 1:** This has been added.

2. Abstract, line 25: please include the number of PAH patients included in this study

**Response 2:** This has been added.

3. Introduction, line 43: specify that keyhole mapping is possible because of the 3D radial k-space trajectory used

**Response 3:** We have added this detail.

4. Methods, line 87: please rephrase this sentence to mention the physiology that the oscillation measurements reflect

**Response 4:** We have added this information.

5. Methods, line 91: please explain why you could not compare oscillation mapping to imaging techniques such as DCE-MRI and PREFUL?

**Response 5:** We have added some justification of this.

6. Methods, ‘Human subjects’: consider renaming this section ‘Participants’ to align with the results section

**Response 6.** We have edited the naming of this section.

7. Methods, ‘Binning algorithms’: mention which binning method ref 9 used

**Response 7:** Reference 9 used a similar peak-finding algorithm to that used by Reference 8. We have added this information in the methods.

8. Methods, line 201 – 202: cut down the amount of words in these equations and replace with symbols where possible

**Response 8:** We have reduced the number of words in equations as suggested.

9. Methods, line 222: were you comparing the mean or median values from the maps? Why didn’t you compare oscillation phase between the groups?

**Response 9:** We compared the mean value, which we have now noted in the methods (Note that using the median provides very similar results to using the mean). For this revision, we additionally compared phase metrics (mean, median, standard deviation, and interquartile range), none of which were different across participant groups.

10. Results: did you look at the map heterogeneity and how this compares between the groups?

**Response 10:** For this revision, we have added information about the coefficient of variation for the different oscillation methods, showing that the 2-key, mean scaled method had the lowest coefficient of variation and the multi-key method the highest. We also examined difference in standard deviation of oscillation amplitude across disease groups, and showed that standard deviation was largest for SSc participants. These considerations have been added to the methods and results.

11. Figures 2 and 3: it would perhaps be helpful to include a Bland-Altman plot alongside each correlation plot.

**Response 11:** Bland Altman plots have been added to figures 2 and 3.

12. Figure 2: why do B and C present the ratios in units of percentage but not A?

**Response 12:** Standard procedure within the Xe-MRI field has been to report RBC/Membrane in absolute units and to report RBC/Gas and Membrane/Gas ratios as (%). This is largely owing to the values measured, where RBC/Membrane typically has values between 0.1 and 0.6, while RBC/Gas and Membrane/Gas have values 2 orders of magnitude lower.

13. Figures 6 and 7: I find the colour maps used for the 2-key oscillation amplitude maps a little hard to interpret. Consider using the same colour map as is used for the multi-key method or a map with a larger dynamic range.

**Response 13.** We have updated images to have different colormaps that we believe have improved interpretability. We prefer to use different color maps for each of the methods to emphasize that the maps were generated using different methods.

14. Figure 9: please explain the outlier in B

**Response 14:** We have generated a new version of this figure (Now Figure 10) based on reviewer comments, and it no longer has such an egregious outlier. There is still an outlier, but this does not have an obvious explanation. For example, SNR of both scans is high, the global oscillation amplitude was very similar for both scans. Oscillation quality was slightly lower (graded as 3), but there were other scans with much worse oscillation quality.

15. Discussion: please add an interpretation of the high oscillation amplitudes observed in SSc patients

**Response 15:** We have added a discussion of the likely meaning of elevated oscillation amplitude in SSc patients.

16. Discussion, line 459: did you look at the correlation of oscillation amplitude with age? In most literature, oscillation amplitude is not correlated with age

**Response 16.** We did not look at this correlation for several reasons, the largest being that we did not have a range of ages in our healthy volunteers over which to examine oscillation amplitude. Our healthy volunteers were all between 25 and 35, so we would be unable to assess correlations with age. Mummy et al. (Front Med 2024) suggest that RBC oscillation amplitude does increase with age, so it is possible that healthy volunteers and SSc participants are not as different as our data would indicate. However, differences between disease states are used to illustrate features of the different technical methodology as opposed to making a claim to clinical utility, so we feel that this is only a minor limitation.
