# Supplementary material for: Optimizing reliability of RBC signal oscillation measures from hyperpolarized 129Xe MRI
Source: PLoS One. 2025 Aug 26;20(8):e0331113. doi: 10.1371/journal.pone.0331113 (PMC12380273; doi:10.1371/journal.pone.0331113)
Supplement: Attachment — Submitted filename: Response_to_Reviewers_Revision2.docx [file pone.0331113.s002.docx]

We thank the reviewers for their careful review of our revisions. We have addressed the one remaining minor comment. All changes in response to this reviewer comment are tracked and commented using the shorthand R for reviewer and C for comment.

Reviewer Comments:

Reviewer 1:

1. Line 538: I don’t follow why the phase maps will be “strongly dependent on the timing of the cardiac cycle with respect to the scan”. To my understanding, the phase is independent of the scan timing as the phase would only be dependent on the timing of the voxel-wise RBC signal maximum relative to the global peak RBC signal maximum. Consider clarifying or removing.

Response: This is a good point. To avoid confusion about this point that is only ancillary to the manuscript’s main points, we have removed the offending sentence.

Other Edits in the Revised Version:

1. In Acknowledgements: Ms. Jemima Pilgrim-Morris just received her PhD, so we have edited “Ms. Jemima Pilgrim-Morris” to “Dr. Jemima Pilgrim-Morris”.
2. References 10 and 17 were duplicates. We have removed this duplicate reference, and the bibliography and numbering of citations has been updated accordingly. All other references have been reviewed for accuracy.
